# Detection of early-stage lung cancer in sputum using automated flow cytometry and machine learning

**DOI:** 10.1186/s12931-023-02327-3

**Published:** 2023-01-21

**Authors:** Madeleine E. Lemieux, Xavier T. Reveles, Jennifer Rebeles, Lydia H. Bederka, Patricia R. Araujo, Jamila R. Sanchez, Marcia Grayson, Shao-Chiang Lai, Louis R. DePalo, Sheila A. Habib, David G. Hill, Kathleen Lopez, Lara Patriquin, Robert Sussman, Roby P. Joyce, Vivienne I. Rebel

**Affiliations:** 1 Bioinfo, Plantagenet, ON Canada; 2 bioAffinity Technologies, 22211 W I-10, Suite 1206, San Antonio, TX 78257 USA; 3 Department of Medicine, Icahn School of Medicine at Mount Sinai, New York, NY USA; 4 South Texas Veterans Health Care System (STVHCS), Audie L. Murphy Memorial Veterans Hospital, San Antonio, TX USA; 5 Waterbury Pulmonary Associates LLC, Waterbury, CT USA; 6 Radiology Associates of Albuquerque, Albuquerque, NM USA; 7 Present Address: Zia Diagnostic Imaging, Albuquerque, NM USA; 8 Atlantic Respiratory Institute, Summit, NJ USA; 9 Precision Pathology Services, San Antonio, TX USA

**Keywords:** Sputum, Automated flow cytometry, Machine learning, Porphyrin, Early-stage lung cancer

## Abstract

**Background:**

Low-dose spiral computed tomography (LDCT) may not lead to a clear treatment path when small to intermediate-sized lung nodules are identified. We have combined flow cytometry and machine learning to develop a sputum-based test (CyPath Lung) that can assist physicians in decision-making in such cases.

**Methods:**

Single cell suspensions prepared from induced sputum samples collected over three consecutive days were labeled with a viability dye to exclude dead cells, antibodies to distinguish cell types, and a porphyrin to label cancer-associated cells. The labeled cell suspension was run on a flow cytometer and the data collected. An analysis pipeline combining automated flow cytometry data processing with machine learning was developed to distinguish cancer from non-cancer samples from 150 patients at high risk of whom 28 had lung cancer. Flow data and patient features were evaluated to identify predictors of lung cancer. Random training and test sets were chosen to evaluate predictive variables iteratively until a robust model was identified. The final model was tested on a second, independent group of 32 samples, including six samples from patients diagnosed with lung cancer.

**Results:**

Automated analysis combined with machine learning resulted in a predictive model that achieved an area under the ROC curve (AUC) of 0.89 (95% CI 0.83–0.89). The sensitivity and specificity were 82% and 88%, respectively, and the negative and positive predictive values 96% and 61%, respectively. Importantly, the test was 92% sensitive and 87% specific in cases when nodules were < 20 mm (AUC of 0.94; 95% CI 0.89–0.99). Testing of the model on an independent second set of samples showed an AUC of 0.85 (95% CI 0.71–0.98) with an 83% sensitivity, 77% specificity, 95% negative predictive value and 45% positive predictive value. The model is robust to differences in sample processing and disease state.

**Conclusion:**

CyPath Lung correctly classifies samples as cancer or non-cancer with high accuracy, including from participants at different disease stages and with nodules < 20 mm in diameter. This test is intended for use after lung cancer screening to improve early-stage lung cancer diagnosis.

*Trial registration* ClinicalTrials.gov ID: NCT03457415; March 7, 2018

**Supplementary Information:**

The online version contains supplementary material available at 10.1186/s12931-023-02327-3.

## Background

Early detection of lung cancer through screening can increase survival and reduce morbidity [1, 2]. The US and regions of the UK recommend annual low-dose computed tomography (LDCT) screening for high-risk individuals [3]. Although LDCT is very sensitive (93.8%) in detecting cancerous pulmonary nodules [4], its specificity is much lower (73.4%) because nodular images can be the result of various non-cancerous processes [5]. A positive LDCT therefore requires follow-up tests to determine if the nodule is malignant [6]. These medical procedures have inherent morbidity and mortality risks and can impose a serious burden on screening participants [7], while associated costs represent significant financial burdens to patients [8] and society [9, 10].

Efforts are underway to develop non-invasive tests that can be used after LDCT to improve screening’s predictive value [11, 12] or as stand-alone tests to identify people who should undergo screening [11, 13]. These tests aim to reduce unnecessary medical procedures while identifying those with lung cancer at an early stage. Sputum is easily accessible lung material that contains a variety of leukocytes and exfoliated bronchial epithelial cells [14], including premalignant and malignant cells in patients with lung cancer [15]. We have previously reported on a slide-based microscopy assay that classified cancer and non-cancer patients using sputum stained with tetra(4-carboxyphenyl)porphyrin (TCPP) [16]. Although 81% accurate, reading slides was time-consuming, subject to observer bias and could potentially miss key events by not evaluating the entire sample. We now report on a high-throughput approach using automated flow cytometry (FCM) instead of microscopy. FCM allows us to interrogate the entire sputum sample using TCPP and a panel of antibodies to capture cancer-predictive features in sputum. The field of automated FCM analysis has produced powerful software tools [17–19] that match or exceed human expertise in identifying cell populations of clinical importance [20]. We adapted these tools to create an automated FCM sputum analysis platform, thereby eliminating potential operator bias [21].

Automated FCM techniques have been combined with machine learning approaches to distinguish leukemias from non-neoplastic cytopenias [22] and for biomarker discovery [23]. We hypothesized that the same combination of approaches could be used to identify sputum features indicative for lung cancer in high-risk patients. Our objective was to develop an assay (referred to as CyPath Lung) that combines automated FCM data analysis of induced sputum samples with machine learning techniques to classify sputum samples as cancer or non-cancer. CyPath Lung is intended for use following screening to improve diagnosis of early-stage lung cancer.

## Methods

### Collection sites

Participants were identified and enrolled at a physician’s or study coordinator’s office at one of the following sites: Atlantic Health System, NJ; Mt. Sinai Hospital, NY; Radiology Associates of Albuquerque, NM; South Texas Veterans Healthcare System, TX; and Waterbury Pulmonary Associates, CT. Each site had received institutional approval to participate in the study. Samples were collected from April 2018 till November 2019 (LSRII sample set) and from July 2020 till November 2021 (Navios sample set).

### Participant information

Participants (males and females) were enrolled in one of two groups. The non-cancer group included participants (aged 52–79) who were either current smokers with a smoking history of at least 20 pack-years, or current non-smokers with a smoking history of at least 20 pack-years, who quit smoking within the past 15 years. The exceptions were two participants: one had quit smoking 26 years ago and one had smoked for 11.5 pack years. Most participants in the non-cancer group had received an LDCT result or other form of imaging that was not suspicious for cancer, and they were advised to return for LDCT screening in 12 months. In a few cases, participants initially placed in the non-cancer group underwent a follow-up LDCT, PET/CT or a biopsy. These participants were followed until their health status was confirmed. If they were diagnosed with lung cancer, they were switched to the cancer group.

Each participant in the cancer group had been evaluated by a physician as highly suspect of having lung cancer based on medical history and LDCT or other imaging results. The diagnosis was confirmed by biopsy after a sputum sample was provided. The exception was a patient who had developed a new nodule of 24 mm and who was too fragile to undergo biopsy. If biopsy showed no cancer, the participant was switched to the non-cancer group. There was no limitation of age or smoking history for enrollment in the cancer group.

For each participant we collected the following demographic data: gender (male or female); age (years); ethnicity (Hispanic/Latino or non-Hispanic/Latino); and race (American Indian/Alaska native; Asian; Black/African American; native Hawaiian/other Pacific islander; White; other). Data on smoking history was collected, as well as data on comorbidities (asthma, COPD, emphysema, chronic bronchitis) and previous cancer history. All participants needed to be willing to provide a primary care physician’s contact information and agree to have medical information released if requested. Exclusion criteria included the presence of severe obstructive lung disease and inability to cough with sufficient exertion to produce a sputum sample, angina with minimal exertion, and pregnancy.

### Sample collection

Sample donors were trained on how to use the acapella assist device (Smiths Medical, St. Paul, MN), and expel their sample by coughing into a specimen cup, repeating this procedure at home for three consecutive days and storing their specimen cup in a refrigerator. Sample donors did not report experiencing any adverse events related to the sample collection procedure. Within one day after collection was completed, the sample was shipped overnight to the bioAffinity laboratory where further processing and FCM analysis took place by technicians blinded to the origin of the sample as well as the clinical information of the donor.

**Fig. 1:**
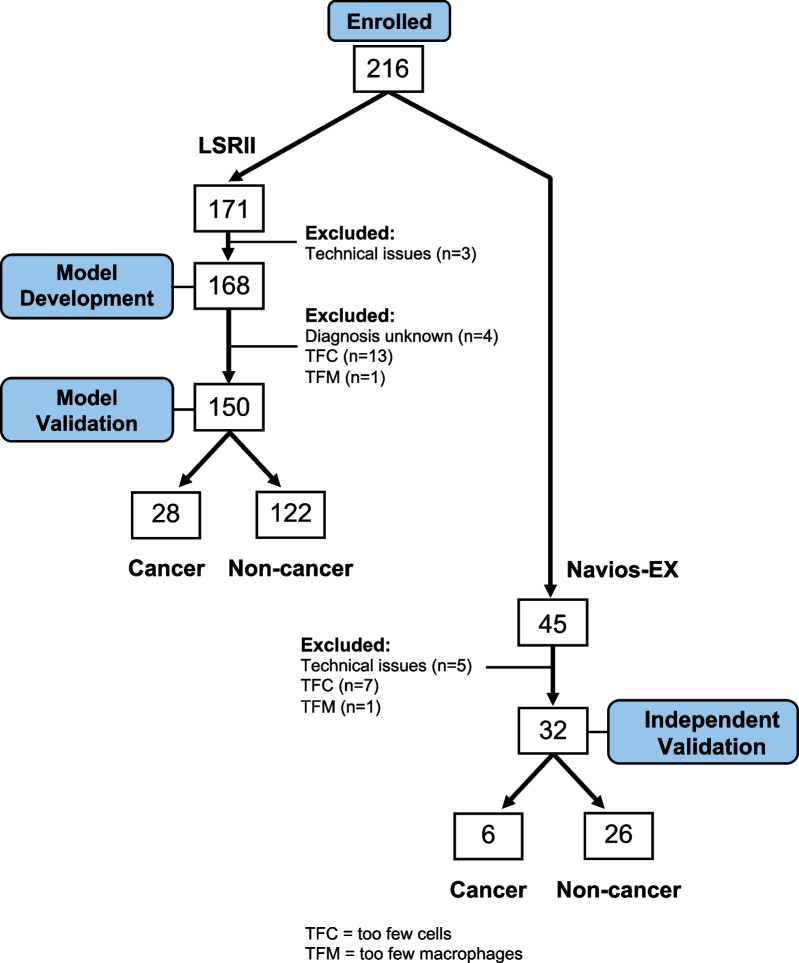
Utilization of Sputum Samples. Of the 171 samples run on the LSRII that were originally considered (136 non-cancer; 31 cancer; 4 with unconfirmed health status), 168 samples were used for model building and analysis pipeline development. This included four samples for which we did not have a definitive disease status because the addition of unlabeled samples had been shown to be helpful in model building [24]. In addition, 14 samples flagged as ineligible based on cell counts (see below) were also used in the model development to better capture the distribution of the underlying data and help make generalization of the model more robust to sample noise. Three samples could not be used at all due to technical problems during acquisition. One hundred and fifty samples were ultimately used for the model validation phase (122 non-cancer; 28 cancer). Eighteen of the 168 samples were omitted: thirteen included too few cells for an accurate analysis, one included too few alveolar macrophages thereby failing to confirm it as a lung sample, and four samples were excluded because their cohort status could not be confirmed. An independent validation of the automated analysis was performed with 32 new samples. Participants adhered to the same enrollment criteria, and samples were processed with the same protocol as the previous sample set. Although a different flow cytometer (Navios EX) was used to run the second set of samples, the same model and coefficients were used to analyze the data for both instruments

A set of 171 sputum samples was analyzed on the LSRII flow cytometer. One hundred and sixty-eight of the LSRII sample set were used for training and testing the model, as well as for developing the analysis pipeline. The final model was then validated on 150 LSRII samples that passed quality control. A second set of 45 samples was analyzed on the Navios EX. Thirty-two passed quality control and were used to independently measure the generality of the model/analysis pipeline by excluding a possible dependency on a particular flow cytometer or team of researchers. See Fig. 1 for more details.

This included four samples for which we did not have a definitive disease status because the addition of unlabeled samples had been shown to be helpful in model building [24].

### Sample size considerations

Enrolment for the LSRII data set continued until sufficient cancer samples were collected to build a robust model for automated analysis. For test development and model training, we needed enough cancer samples in order to create subsets of samples through repeated randomization that would allow us to evaluate cancer predictor selection without repeatedly ending up with the same small number of cancer samples driving the model fitting. With 28 out of 150 samples being a cancer sample (~ 19%), we would be able to create  > 3 million different training sets, each consisting of 100 samples (including 20 cancer samples, 20%). Three million different training sets are more than enough to test a wide range of potential parameters without a strong selection bias, while maintaining the cancer / non-cancer sample ratio of the entire sample set.

The Navios data set represents the number of samples recruited between establishing the analysis pipeline and the start of drafting this manuscript.

### Sputum processing

Sputum was dissociated and labeled using recently published protocols [25, 26]. Briefly, sputum samples were incubated with a mixture of 0.1% dithiothreitol and 0.5% *N*-acetyl-l-cysteine for 15 min at room temperature and neutralized with Hank’s Balanced Salt Solution (HBSS). Cells were then filtered through a 100-micron nylon strainer, washed and re-suspended in HBSS. Total cell yield was determined using trypan blue exclusion.

A small aliquot of cells was set aside for use as controls while the majority was divided into two tubes for the main analysis. Both tubes were labeled with Fixable Viability Stain 510 (FVS510) and CD45-PE. One tube, the “blood tube”, received CD66b-FITC (to identify granulocytes), CD3-Alexa-Fluor-488 (T-cells), CD19-Alexa-Fluor-488 (B-cells) and CD206-PE-CF594 (macrophages). In the other tube, the “epithelial tube”, cells were labeled with the epithelial cell markers pan-cytokeratin (Pan-CK)-Alexa-Fluor-488 and EpCAM-PE-CF594. Cells were incubated for 35 min on ice, protected from light. After washing with HBSS, cells were fixed and stored on ice until the next day, when a TCPP solution (20 µg/mL) was added (3.3 × 10^6^ cells/ml; 1:1 v/v) for 1 h. Cells were washed twice and kept on ice and protected from light until analysis.

### Flow cytometry

Sputum samples were acquired on a BD LSRII flow cytometer (BD Biosciences) equipped with four lasers (405 nm, 488 nm, 561 nm, and 633 nm) or on a Navios EX (Beckman Coulter Life Sciences) equipped with three lasers (405 nm, 488 nm and 638 nm). The settings used on each flow cytometer had been previously shown to generate similar sputum profiles [25].

## Results

### Automated flow cytometric selection of viable single cells

**Fig. 2:**
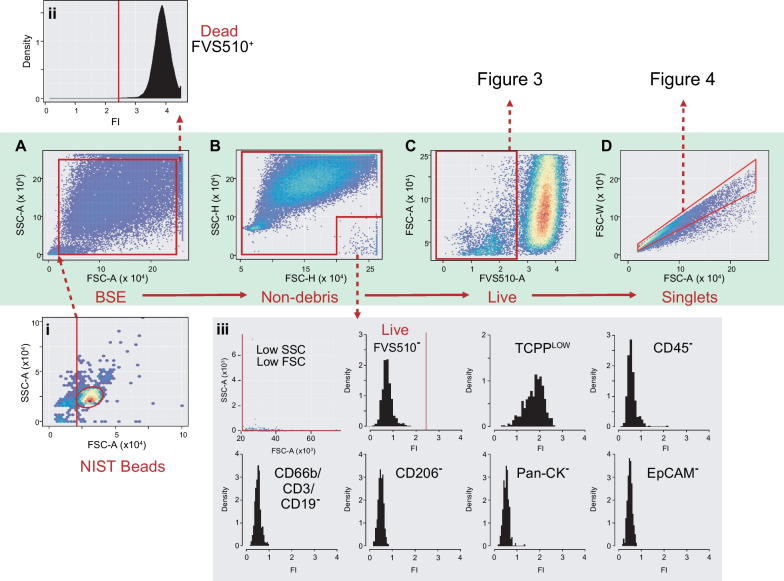
Automated gating of FCM data. **A** Bead size exclusion gate (BSE) parameters are set on the entire sputum sample with the bottom left threshold derived from automated NIST bead flowClust analysis (i inset). The top right corner of the BSE gate is set at 2.5 × 10^5^ based on the observation that events above that threshold on both forward (FSC) and side (SSC) scatter area are dead cells (ii inset; FVS510 viability histogram). **B** In some samples, events appear in the lower righthand corner of FSC-H vs SSC-H plots. These events are gated out to avoid including them as live, viability marker negative, TCPP dull cells as shown in (iii). **C** A viability threshold is set on the Non-debris events based on FVS510 fluorescence (details in Fig. 3 and Additional file 1). **D** A singlets gate is then set on viable events (details in Fig. 4 and Additional file 1). Viable singlets are used for downstream numerical analysis. FI: Fluorescence Intensity

The first stage of the CyPath Lung assay is the automated FCM identification of viable single cells (Fig. 2). The FCM component of the test consists of two assay tubes, one labelled with blood cell markers and one with epithelial cell markers [25]. Cells in both tubes were also labeled with anti-CD45 antibodies which selectively bind leukocytes, a viability dye to eliminate dead cells, including squamous epithelial cells (SECs) [27], and TCPP to identify cancer or cancer-associated cells [28]. Fluorescence intensities from antibody and TCPP staining were used exclusively for downstream numerical analysis.

Each sample run included polystyrene beads of known diameter (5–30 µm NIST beads), compensation tubes for each fluorochrome channel used, unstained sputum, and an antibody isotype sputum control. Each tube corresponded to a single Flow Cytometry Standard (FCS) file that contained sample metadata and per event values for each light and fluorescence channel acquired plus a Time parameter recorded as tubes are run.

As shown in Fig. 2A, the first step was to restrict events based on forward (FSC-A) and side (SSC-A) scatter area, both reasonable surrogates for cell size [29]. A two-dimensional cluster gate identified the dominant peak of 5 µm NIST beads in FSC-A vs SSC-A (Fig. 2A(i)). The lower FSC-A limit of the bead cluster was set as the minimum sample FSC-A to exclude small particulates and debris. Upper limits of 2.5 × 10^5^ were set on both FSC-A and SSC-A since events above those thresholds were found to be dead (FVS510^+^) cells (Fig. 2A and A(ii)).

Events within the bead size exclusion (BSE) gate were then restricted to exclude a population with unusual FSC and SSC height profiles (Fig. 2B) and a staining profile that might result in their inappropriate inclusion in downstream analyses (Fig. 2B(iii)). Exclusion of unusual-looking populations is warranted in general [30]. In our case, it is important to exclude these spurious events to avoid including them in viable cell counts since the decision to include a sample for full analysis depends on total viable singlets.

**Fig. 3:**
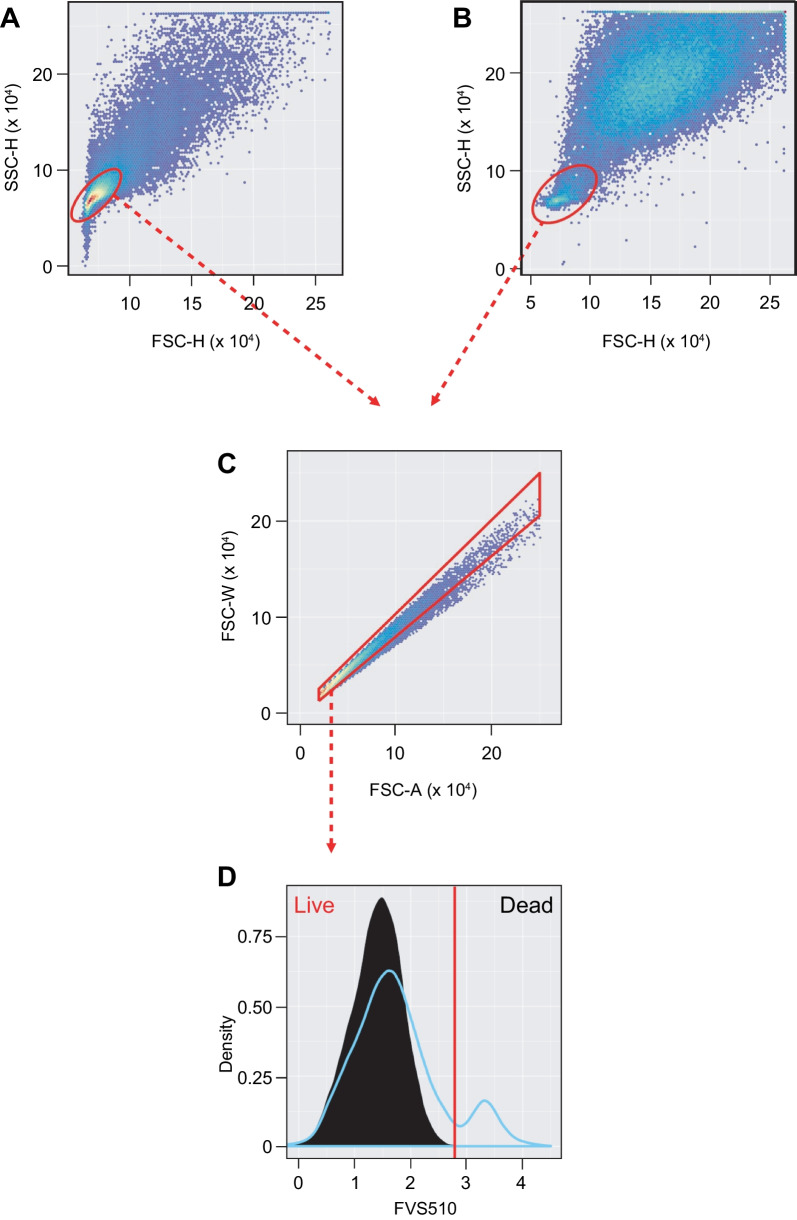
Heuristics-guided Viability Gate Setting. **A** Set a temporary flowClust gate on Non-debris events in FSC-H vs SSC-H to retain mostly live cells for eventual FVS510 tail gating (“core viable gate”). **B** For samples with < 10% events in the core viable gate, rerun flowClust more inclusively by increasing “quantile” parameter to 0.99. **C** Set a temporary singlets gate on core viable events, forcing the capture of the upper diagonal by setting the top right point to 2.5 × 10^5^ on both axes. **D** A tail gate with 10% tolerance is set automatically on the core viable singlets (black histogram). Shown in blue is the full Non-debris FVS510 profile for comparison. The red bar indicates the viability gate cutoff. Viable events are to the left of the threshold. All temporary gates are removed once the threshold is determined

Cells below the threshold for FVS510-positivity were retained (Fig. 2C). Heuristics based on subpopulations most likely to contain viable singlet cells (i.e., relatively small in area and height in light scatter channels) were used to guide its positioning (Fig. 3 and Additional file 1).

**Fig. 4:**
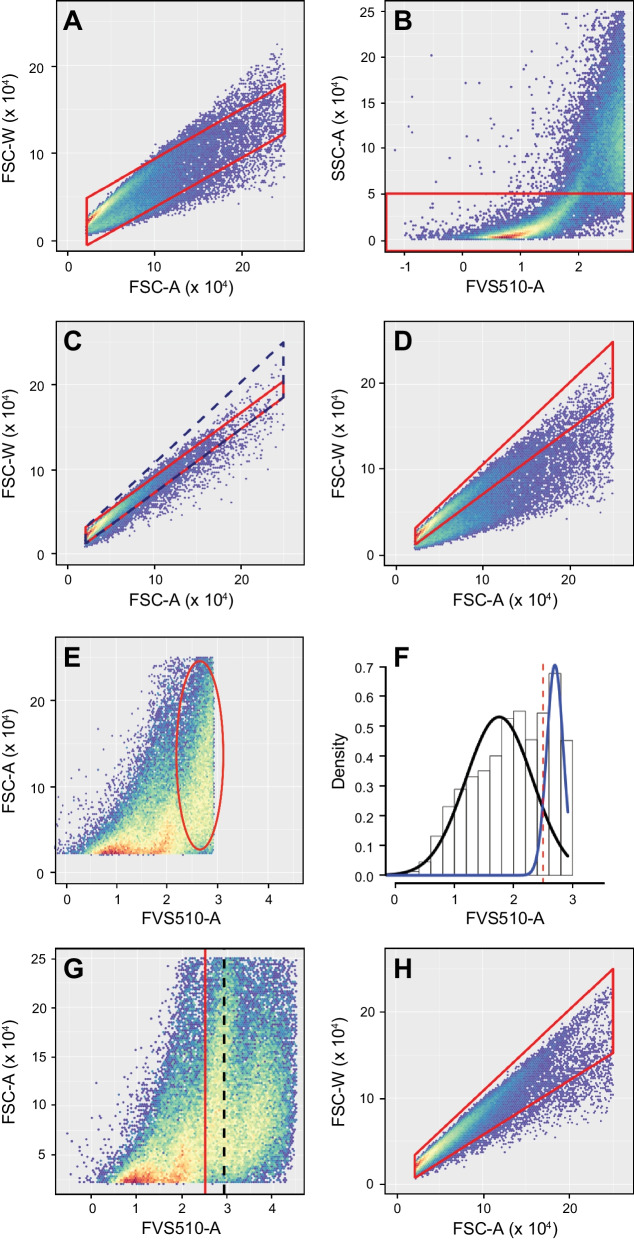
Heuristics-guided Singlets Gating. **A** In some cases, cells with intermediate FVS510 signal and high SSC-A throw off singlets gating on the full viable cell population (e.g., lower left corner below zero or top left point higher than bottom right). **B** A temporary gate is set on SSC-A to exclude problematic events above 5 × 10^4^. **C** A singlets gate is fitted to the restricted population (red polygon) and adjusted to include the upper diagonal by setting the upper right corner to 2.5 × 10^5^ on both axes (navy dashed polygon). **D** Temporary gates are removed and the tweaked singlets gate (red polygon) applied to the full viable population (compare the adjusted polygon in **D** to the original red polygon in **A**. **E** In some cases, a population representing > 10% of singlets (red oval) lies between 2.5 (logicle scale) and the viability threshold. **F** Population mixture analysis highlights the difference in signal distribution of the rightmost population identified by the oval in **E** (blue curve) relative to the bulk of the events left from the oval in **E** (black curve) and suggests a natural cutoff at 2.5 (dashed red line) in these unusual cases. **G** The adjusted viability cutoff (red line) replaces the one found by automated tail gating (dashed black line). **H** Finally, a new singlets gate for the refined viable cell population is calculated (red polygon). **A**–**D** are from a different patient sample than **E**, **F** to illustrate the heuristics applied in singlets gating

A “singlets” gate (FSC-Area vs FSC-Width) excluded cell doublets or small aggregates (Fig. 2D). In some samples, high SSC-A cells are included in the viable cell population and distort the results of the singlets gating algorithm. This can be corrected by fitting the gate to a temporary subpopulation excluding most of these high SSC-A events (Fig. 4A–D). In other samples, two populations can be seen within the viable cell gate, one with low FVS510 staining and the other just below the viability threshold and with a high side scatter profile (Fig. 4E–H). The correction involves resetting the viability gate and fitting the singlets gate to the more restricted viable population (see Additional file 1 for more details). Light scatter and fluorescence signal values were recorded for each single event and used for downstream model development and validation.

### Development of the CyPath Lung cancer/non-cancer classifier

The second stage of the CyPath Lung assay is the analysis of light scatter and fluorescence signals from the viable single cells identified in the first stage by automated FCM. Logistic regression models describe a relationship between predictor variables and a categorical (in our case binary cancer/non-cancer) response variable. We measure our model’s performance by comparing its prediction of whether a sample is cancer or non-cancer (the response variable) to the known cancer/non-cancer status of the samples. Stepwise regression is a supervised machine learning process by which potentially predictive variables are added and removed and the resulting model examined for goodness of fit (see Additional file 1 for details). Demographic and clinical data (see Table 1) were included as potential predictors. Age was the only clinical parameter repeatedly rated as significant during forward and reverse stepwise regression.

**Table 1:** Patient characteristics of LSRII validation samples

Characteristic		Non-cancer, n = 122	Cancer, n = 28
Patient demographics			
Age—years	median (range)	65 (53-75)	73 (54–79)
Male	n (%)	57 (46.7)	21 (75.0)
Female	n (%)	65 (53.3)	7 (25.0)
Race			
White	n (%)	110 (90.2)	25 (89.3)
Non-white	n (%)	12 (9.8)	3 (10.7)
Ethnicity			
Hispanic	n (%)	15 (12.3)	8 (28.6)
Non-Hispanic	n (%)	104 (85.2)	18 (64.3)
Not available	n (%)	3 (2.5)	2 (7.1)
Smoking Status			
Never	n (%)	0 (0)	1 (3.6)
Former	n (%)	69 (56.6)	15 (53.6)
Pack years	mean (SD)	56.1 (24.3)	53.3 (36.3)
Current	n (%)	53 (43.4)	12 (42.9)
Pack years	mean (SD)	55.2 (26.5)	51.8 (14.1)
Comorbidities			
COPD	n (%)	81 (66.4)	13 (46.4)
Emphysema	n (%)	23 (18.9)	6 (21.4)
Asthma	n (%)	16 (13.1)	4 (14.3)
Bronchitis	n (%)	7 (5.7)	3 (10.7)
Cancer	n (%)	17 (13.9)	3 (10.7)

n: number of samples; SD: standard deviation

**Fig. 5:**
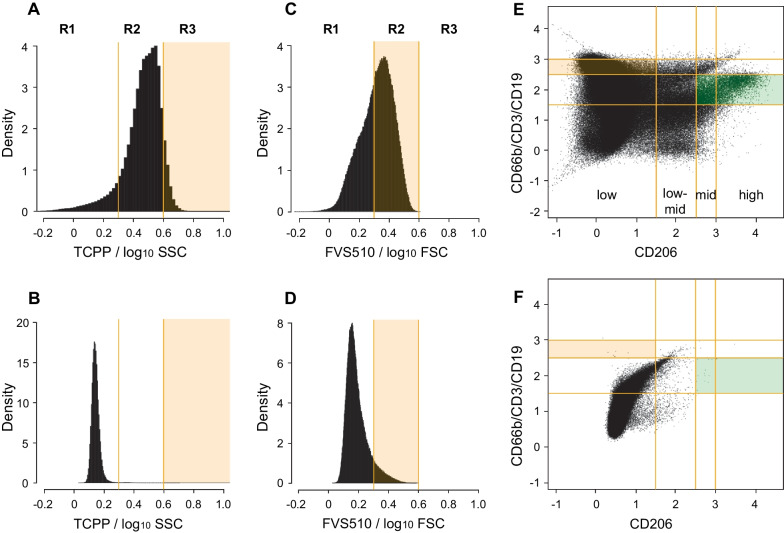
Model variables and sample quality metrics from FCM. **A** Region R3 (shaded) counts represent viable singlets with high TCPP signal relative to side scatter (log10-transformed). Compare **A** to unstained control tube in **B**. **C** Events in region R2 (shaded) are below the viability threshold but nevertheless have relatively high FVS510 signal relative to forward scatter (log10-transformed). Compare **C** to unstained control tube in **D**. **E** The tan-shaded area contains non-macrophage (CD206^low^) leucocytes (CD66b/CD3/CD19^mid^). The area shaded in green (CD206^mid/high^ CD66b/CD3/CD19^low−mid^) contains lung macrophages required for the sample to be considered to adequately sample the lung environment. Compare **E** to unstained control sample in **F**. All panels are from the same illustrative sample

Based on our earlier slide-based assay results, we anticipated that smoking history (or correlated factors like age) and TCPP signal density (as opposed to fluorescence intensity itself) would be important predictors [16]. We therefore divided the fluorescence signals of all channels by log_10_ FSC-A or log_10_ SSC-A and partitioned the resulting density distribution into three regions (< 0.25, 0.25–0.6, > 0.6, Fig. 5A–D). Two such density signals proved informative for the classifier: TCPP/log_10_SSC-A (Fig. 5A, region 3 [R3]) and FVS510-A/log_10_FSC-A (Fig. 5C, region 2 [R2]). The predictive value of TCPP/log_10_SSC-A signal density was not imposed upon the stepwise regression but emerged spontaneously. The fact that FVS510-A signal density was also found to be informative is interesting and may be related to the fact that apoptotic cells can take up this dye at intermediate levels [31]. No other single blood or epithelial fluorescence signal density was robustly and repeatedly identified as a predictor.

Combinations of lineage markers can identify subpopulations that single markers alone may not capture. Careful examination of patient samples revealed complex patterns of lineage marker expression in blood and epithelial tubes. Although there were differences between the non-cancer and cancer groups [26], we could not directly identify any subpopulation independently predictive of cancer by gating. Consequently, we decided to use a numerical approach to the analysis of pairwise markers by partitioning fluorescence based on signal distribution in blood (Fig. 5E, F) and epithelial tubes (data not shown). Signal intensity on the logicle scale was quantized into low (< 1.5), low-mid (1.5–2.5), mid (2.5–3), and high (> 3) windows. Events per 10,000 were tabulated for each area of the resulting 4 × 4 grid of CD206 vs CD3/CD19/CD66b (blood tube) or EpCAM vs Pan-CK (epithelial tube; not shown). One area in the blood signal intensity grid was found to be informative for the model (tan-shaded rectangle; low for CD206 and mid-level for CD3/CD19/CD66b, Fig. 5E). This population may indicate the presence of immune or inflammatory processes in the lung [32].

Once we had reduced the list of potential predictors to those that were most promising, we tested for pairwise interactions between them. One interaction proved informative: adding a negative value proportional to [age x number of events in FVS510-A/log_10_FSC-A R2] improved the classifier’s performance. Our interpretation of this interaction term is that it serves to moderate a possibly age-related accumulation of stressed cells in the non-cancer patient group as a consequence of smoking or health history [33].

### Running the CyPath Lung assay pipeline

**Fig. 6:**
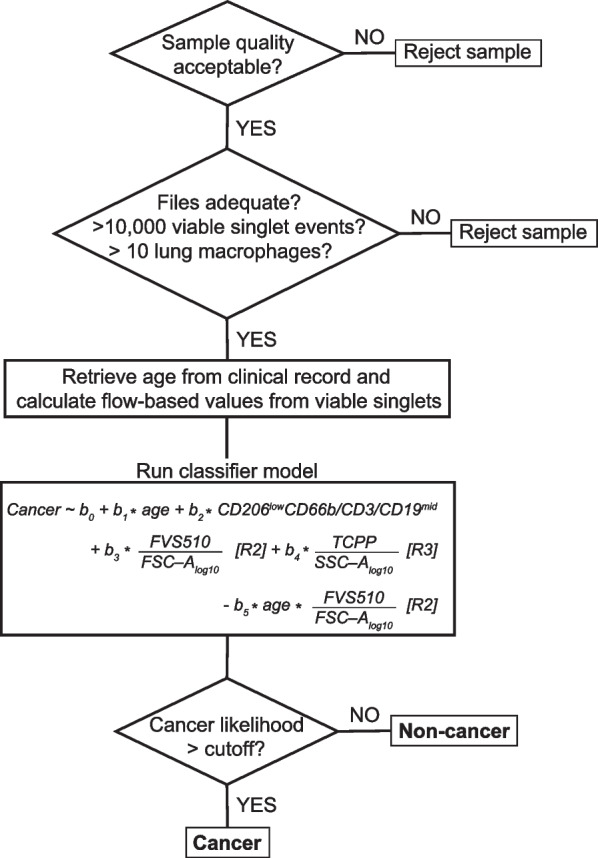
CyPath Lung data processing pipeline. The schematic represents the following main elements: QC measures (top two diamonds), retrieval of the data and running the model (two squares in the middle) and final determination whether the sample is likely to be cancer or not (bottom diamond)

Having developed the two stages of the CyPath Lung assay, we could now assemble the full pipeline, including quality control steps, determination of predictive variable values, and classification of samples (Fig. 6).

Sample quality assessment begins by ensuring that the data file for each collection tube is readable and that its encoded data matrix is complete. Next, the Time signature is used to examine fluorescence channels in each tube and to remove anomalies in the flow rate arising from bubbles or clogs during sample acquisition [34]. Fluorescence compensation tubes are used to derive the compensation matrix de novo (as opposed to using the compensation matrix encoded in the sample file metadata). Fluorescence signal is compensated and transformed to the logicle scale [35] to produce the sample data matrix used by the automated FCM gating to isolate viable singlet events. In order to have confidence in the downstream numerical analysis, given potentially small numbers of events in some analysis windows, we required that samples contain at least 10,000 viable singlets. We also required that at least 10 cells be present in the green-shaded area of Fig. 5E in which we find lung macrophages (CD206^mid&high^CD3/CD19/CD66b^low−mid^) [36] to confirm that the sputum sample originated in the lung.

The next step in the assay pipeline (Fig. 6) is to supply age in years and flow-based values from viable singlets to the classifier model. The likelihood of having cancer depends on four variables: age, number of events per 10,000 viable singlets (per 10K) with TCPP/log_10_SSC-A in Region 3 (Fig. 5A, R3), number of events per 10K with FVS510-A/log_10_FSC-A in Region 2 (Fig. 5C, R2), and number of events per 10K in the CD206^low^CD3/CD19CD66b^mid^ sector (Fig. 5E, tan shading). In addition, the model contains a negative term for the interaction between age and the FVS510 density variable and an “intercept” term (b_0_). The intercept term improves model fitting by not forcing the fitted line through 0 if all the variables are set to zero, but it is not directly interpretable as a biologically meaningful component of the classifier. The values of the coefficients (b_1_, b_2_, b_3_, b_4_, and b_5_) depend on the training set used for model fitting and provide weights for the variables. We did not normalize the data provided to the model in order to make interpretation of the model easier. For example, the model formula tells us that increasing the number of events with high TCPP density increases the likelihood of cancer, consistent with our previous results [16].

**Fig. 7:**
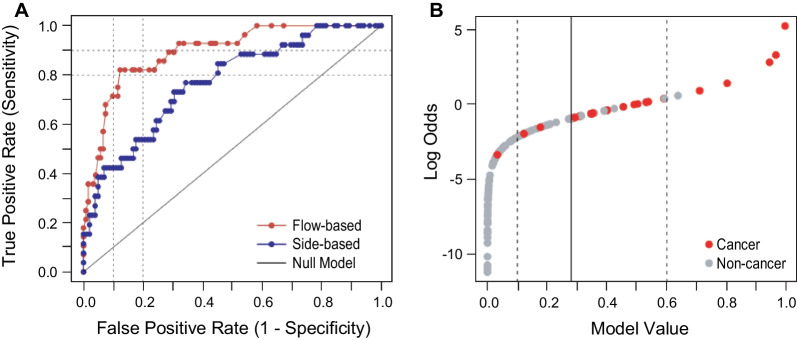
CyPath Lung performance on LSRII samples. **A** Receptor operating characteristic (ROC) curve showing false positive vs true positive rates calculated as the model response threshold was varied (red curve). For comparison, the ROC curve of the previous version of the slide-based CyPath Lung assay [16] is shown in blue. Dashed lines indicate 80% and 90% sensitivity and specificity (1 − False Positive Rate). **B** Based on the ROC curve in **A**, a model value (likelihood of cancer) threshold was set at 0.28 (solid vertical line), corresponding to a sensitivity of 82.1% and sensitivity of 87.7%. The dashed lines indicate levels below which a prediction of cancer is very unlikely (leftmost dashed line) or very likely (rightmost dashed line)

The final step of the pipeline (Fig. 6) is to make the cancer/non-cancer assignment. The model returns a value in the range of [0, 1]. Whether a given sample is classified as cancer depends on having the model return a value greater than a predetermined cutoff. If the value is less than or equal to the cutoff, the sample is classified as non-cancer. A rational cutoff can be selected by stepping through cutoff values between 0 and 1 and measuring the true positive and false positive calls as compared to the known group category at each step. Figure 7A shows the result of this process as a receiver operating characteristic (ROC) curve, with an area under the curve (AUC) of 0.89. The assay achieved its best performance at discriminating cancer from non-cancer at a threshold of 0.28 (Fig. 7B, solid vertical line).

### Performance of CyPath Lung

We evaluated the performance of CyPath Lung for the 122 non-cancer and 28 cancer samples described in Table 1 and for an additional 32 samples (26 non-cancer and 6 cancer; Table 2) processed on a different FCM instrument (Navios EX). The same model with the same coefficients was used for both instruments, but the cutoff for the Navios samples was 0.5, based on the ROC curve for these samples. The results shown in Table 3 demonstrate that CyPath Lung performed very well with sensitivity, specificity, and accuracy all > 80% for the LSRII samples and very similar numbers for the smaller set of Navios EX samples. For both flow cytometry platforms we obtained a very robust negative predictive value (NPV) ≥ 95%.

**Table 2:** Patient characteristics of Navios EX validation samples

Characteristic		Non-cancer, n = 26	Cancer, n = 6
Patient demographics			
Age—years	mean (Range)	65 (52–79)	64 (49–76)
Male	n (%)	15 (57.8)	6 (100)
Female	n (%)	11 (42.3)	0
Race			
White	n (%)	20 (76.9)	5 (83.3)
Non-white	n (%)	2 (7.7)	1 (16.7)
Not available	n (%)	4 (15.4)	0
Ethnicity			
Hispanic	n (%)	5 (19.2)	0
Non-Hispanic	n (%)	17 (65.4)	6 (100)
Not available	n (%)	4 (15.4)	0
Smoking status			
Never	n (%)	0	1 (16.7)
Former	n (%)	14 (53.8)	2 (33.3)
Pack years	mean (SD)	60.1 (27.3)	90 and 25^a^
Current	n (%)	11 (42.4)	3 (50.0)
Pack years	mean (SD)	52.4 (27.7)	75.0 (37.0)
Not available	n (%)	1 (3.8)	0
Comorbidities			
COPD	n (%)	9 (34.6)	3 (50.0)
Emphysema	n (%)	4 (15.4)	1 (16.7)
Asthma	n (%)	1 (3.8)	0
Bronchitis	n (%)	1 (3.8)	0
Cancer	n (%)	1 (3.8)	2 (33.3)
Not available	n (%)	2 (7.7)	0

n: number of samples

^a^Individual values are shown instead of mean (SD)

**Table 3:** CyPath lung performance

	LSRII	LSRII (nodules all < 20 mm)	Navios
Total samples	150	132	32
Cancer	28	13	6
Non-cancer	122	119	26
Sensitivity (95% CI)	0.82 (0.64–0.92)	0.92 (0.67–0.99)	0.83 (0.44–0.97)
Specificity (95% CI)	0.88 (0.81–0.92)	0.87 (0.80–0.92)	0.77 (0.58–0.89)
Accuracy (95% CI)	0.87(0.80–0.91)	0.88 (0.81–0.92)	0.78 (0.61–0.89)
Area under ROC curve (95% CI)	0.89 (0.83–0.96)	0.94 (0.89–0.99)	0.85 (0.71–0.98)
Positive predictive value (95% CI)			
Cancer prevalence in data set	0.61 (0.48–0.72)	0.44 (0.33–0.57)	0.45 (0.27–0.65)
Prevalence reported in high-risk^a^	0.05 (0.03–0.09)	0.06 (0.04–0.92)	0.03 (0.01–0.06)
Prevalence in LDCT positive^b^	0.17 (0.11–0.25)	0.18 (0.12–0.26)	0.10 (0.05–0.19)
Negative predictive value (95% CI)			
Cancer prevalence in data set	0.96 (0.91–0.98)	0.99 (0.94–1.00)	0.95 (0.77–0.99)
Prevalence reported in high-risk^a^	1.00 (1.00–1.00)	1.00 (1.00–1.00)	1.00 (1.00–1.00)
Prevalence in LDCT positive^b^	0.99 (0.99–1.00)	1.00 (0.98–1.00)	0.99 (0.96–1.00)
Positive diagnostic likelihood ratio (PDLR)^c^	6.31	7.08	3.61

^a^0.83% cancer prevalence in NLST 2013 [1]

^b^2.9% cancer prevalence in NLST 2013 LDCT positive cases only

^c^Sensitivity/(1 − specificity) see Pepe et al. [37]

Wilson confidence intervals (CIs) for sensitivity, specificity and accuracy were calculated using BinomCI (“method = Wilson”) from the R package DescTools [38]

Area under ROC curve CIs were determined by bootstrapping using the R package pROC [39]

CIs of the positive and negative predictive values were calculated using the R package bdpv [40] per Mercaldo et al. [41]

The assay also performed remarkably well, with a sensitivity of 92% and specificity of 87% and an area under the ROC curve of 94%, if we restricted the analysis to cases where LDCT detected no nodules or only nodules < 20 mm in diameter (Table 3, “nodules all < 20 mm”). We do not consider the difference in the sensitivity and specificity between the full data set and the subset with nodules < 20 mm significant; however, it is evidence that the test performed equally well for difficult to diagnose individuals with smaller nodules. Furthermore, CyPath Lung performed well for all tumor types represented and at all disease stages, including I and II (Tables 4, 5).

**Table 4:** Performance of CyPath lung by tumor type and stage (LSRII)

Tumor type (Carcinoma)	n (%)	# of cancers correctly predicted	Stage	n (%)	# of cancers correctly predicted
Non-small cell	1 (3.6)	1	I	10 (35.7)	8
Adeno	11 (39.3)	8	II	3 (10.7)	2
Squamous cell	13 (46.4)	11	III	6 (21.4)	5
Large cell	1 (3.6)	1	IV	6 (21.4)	5
Small cell	2 (7.1)	2	NA	3 (10.7)	3

n: number of samples; NA: information not available

**Table 5:** Performance of CyPath lung by tumor type and stage (Navios EX)

Tumor type (Carcinoma)	n (%)	# of cancers correctly predicted	Stage	n (%)	# of cancers correctly predicted
Non-small cell	0		I	3 (50.0)	2
Adeno	3 (50.0)	2	II	0	
Squamous cell	2 (33.3)	2	III	2 (33.3)	2
Large cell	0	0	IV	0	
Small cell	0	0	NA	1 (16.7)	1
NA^a^	1 (16.7)	1			

n: number of samples; NA: information not available

^a^Biopsy was not performed because of comorbidities. However, this patient is treated as having lung cancer due to the presence of a 24 mm nodule and other factors

**Fig. 8:**
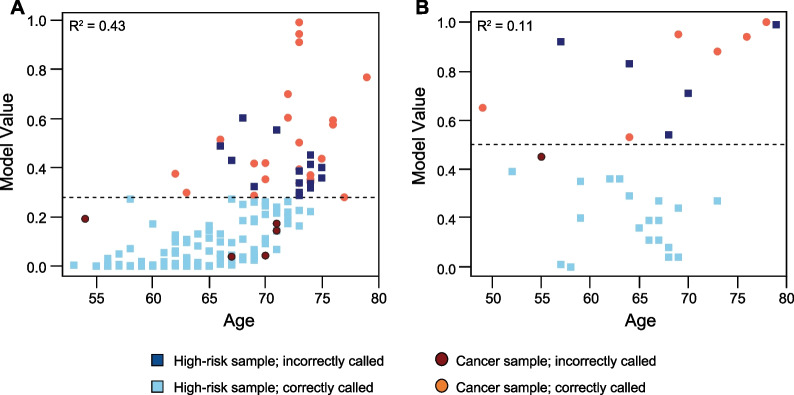
Correlation of age with model value. **A** LSRII samples. **B** Navios EX samples. The dashed lines indicate the cutoff value for the respective sample set above which a sample is diagnosed as cancer

Each of the retained predictors contributed significantly to the model (Wald Test *P* < 0.05) and removing them individually had a negative impact on the ability to correctly classify cancer and non-cancer samples (Table 6). Age is a well-established clinical correlate to lung cancer [42], as it is in our model; nevertheless, the correlation between age and the model value is not overwhelming in either LSRII or Navios EX samples (Fig. 8) with "cancer" called in some younger patients and "non-cancer" in many older ones. In fact, the exclusion of the CD206^low^CD3/CD19CD66b^mid^ signal resulted in as many misclassified samples as the exclusion of age and its interaction with FVS510-A/log_10_FSC-A R2 (Table 6).

**Table 6:** Impact of model predictors on classification

Predictor dropped from model	Cancer called^b^		Non-cancer called^b^		Total (Cancer and Non-cancer)^b^
	Correct	Incorrect	Correct	Incorrect	Incorrect
None^a^	23	5	107	15	20
age^c^	9	19	111	11	30
TCPP/log_10_SSC-A R3	18	10	105	17	27
CD206^low^CD3/CD19CD66b^mid^	20	8	100	22	30
FVS510-A/log_10_FSC-A R2^c^	19	9	107	15	24
age:FVS510-A/log_10_FSC-A R2	18	10	104	18	28

^a^Full model as shown in Fig. 5

^b^150 LSRII samples from Table 1

^c^Including interaction term age:FVS510-A/log10FSC-A R2

## Discussion

To our knowledge, this study is the first that combines automated flow cytometric analysis with machine learning to predict the presence of lung cancer from sputum samples. Sputum as diagnostic material provides a snapshot of the tumor itself, of its microenvironment (ME), and of its field of cancerization (FoC). Expert cytological analysis of sputum can detect cancerous and pre-malignant cells [15], but it is a laborious approach that does not lend itself well to large-scale screening. Automated image processing has been used with some success to capture malignancy-associated changes in cells but is hampered by technical complexity and the low numbers of cells analyzed [43].

The case for moving to a high-throughput, automated flow-based approach combined with machine learning is thus compelling: (a) the assay can be put into routine lab use without requiring expert evaluation of samples or being subject to operator bias; (b) the entire sputum sample can be rapidly analyzed; and (c) numerical analysis can capture complex interactions between lung cancer, ME, and FoC cells which would be difficult for individuals to detect reliably. Our discovery during assay development of the predictive value of viability staining density, for example, suggests a link with apoptosis that merits further study. Our model also indicates that specific immune cell populations may be involved. Neither of these predictors was on our radar before we began model building, but in retrospect the importance of both processes early in cancer development is consistent with previous reports [42, 44].

Earlier versions of CyPath Lung relied solely on the porphyrin to distinguish cancer from non-cancer samples [16]. The current automated flow cytometry-based test leverages viability staining and antibody profiling to capture additional aspects of tumorigenesis. One of the cancer predictors in CyPath Lung reflects an increase in immune cells in the cancer group. Since alterations in the immune system constitute an early response of the body to the presence of a tumor [45], it is possible that CyPath Lung can detect certain cancers before they are detectable by imaging. Others have shown that the performance of a sputum-based test for early lung cancer detection can significantly increase when different types of measurements are combined; for example, cytology with genetic mutations [46] or microRNAs and methylation biomarkers [47]. Although we use one technology platform to measure different cancer-related processes, the additional parameters are likely contributing to the performance improvement from the slide-based assay to the flow cytometry-based assay (Fig. 7). Moreover, the flow cytometry-based assay reads the entire sample, which was also predicted to increase test performance [16].

Nearly 95% of participants in this study fulfilled the criteria for lung cancer screening most recently issued by the US Preventive Services Task Force [48]. Although our study group can be considered a sample from those eligible for lung cancer screening (one of the target populations for CyPath Lung), the sampling was small with minorities being underrepresented, as were females in the cancer groups. Moreover, the cancer prevalence in our study was just below 19% for both data sets, which is considerably higher than in a lung cancer screening population [1] or in a patient group with lung nodules between 7 and 19 mm (the other target population for CyPath Lung) [49]. Another limitation of our study is the lack of long-term follow-up of non-cancer participants to confirm they were indeed lung cancer-free. We intend to conduct a larger prospective clinical trial that addresses these limitations.

In its 2017 Official Policy Statement, the American Thoracic Society (ATS) stated that clinical usefulness of a novel biomarker should be evaluated by estimating the minimal accuracy required for that biomarker [50]. A positive CyPath Lung test may help evaluate intermediate-sized lung nodules in LDCT-positive patients. The minimal accuracy ([sensitivity/(1-specificity] or positive diagnostic likelihood ratio (PDLR)) for CyPath Lung needs to be ≥ [(1-prevalence)/prevalence] × R/(1 − R)] according to the ATS statement. Assuming the threshold (R) – above which invasive follow up would be worthwhile - to be the frequency of cancer (4.8%) in the National Lung Screening Trial (NLST) population with intermediate nodules (7–19 mm in diameter) and using a cancer prevalence of 3.8% in the LDCT-positive population based on data of the NLST, we calculated the PDLR of CyPath Lung should be at least 1.28 [37, 50], which is a threshold met comfortably by our assay (Table 3).

The ATS statement also presents a use case where screening is expanded to include participants currently ineligible for LDCT screening [50]. Using a hypothetical 1/500 prevalence of cancer and a harm threshold of 0.83%, a PDLR of 4.18 is estimated as the minimal accuracy for a useful test, a level met by the larger validation group of CyPath Lung (Table 3, LSRII). Using a hypothetical prevalence of 1/400 instead of 1/500 would yield a PDLR of 3.35, which both validation groups satisfy. When clinical utility is confirmed by future studies, CyPath Lung could serve to expand early lung cancer screening to relatively underserved populations such as younger females and male African American smokers [51, 52].

## Conclusion

CyPath Lung is a non-invasive, sputum-based test for the early diagnosis of lung cancer. It uses a flow cytometric platform to analyze the cellular content of sputum with the analysis being fully automated and thus unbiased. The test is robust to differences in sample handling and processing and captures important predictive factors of early lung cancer carcinogenesis. The test performs well at 82% sensitivity and 88% specificity and achieves comparable performance when applied to an independent set of samples collected on a different flow cytometer. The test is also accurate in early stages (I and II) and in cases with nodules < 20 mm.

## Supplementary Information

**Additional file 1. **Supplemental methods. Detailed description of automated flow cytometry data analysis and classifier development using generalized linear models.

## Data Availability

Flow cytometry data files to reproduce the figures can be found in the FLOW repository (http://flowrepository.org) by accessing the following links: http://flowrepository.org/id/RvFrofFdPABAo3nGTsImVOuDSGrGDnt8PyAF8lc7PbLRmMTOWBbd7iDN0f9LalAG; http://flowrepository.org/id/RvFrTMVZGSin6hn2CH4oG2SZTPCTHni7Rs7vf5HxBzLH6s1sZdwhxDX4jXPxvFm7; http://flowrepository.org/id/RvFr9hmfgMXWnefKQbA9bkupqiEPy0DL6KAJxtpl7ZHnQzFWVU4LWDWojONbbkwD; http://flowrepository.org/id/RvFrF2ln2eH3W4IYYl9BOlaMhUlLd0zgsmxfwuhdzYhK67ve9iMwo1p7AYgUwZnJ. The entire data set used in this study is available from the corresponding author upon reasonable request.

## Abbreviations

BSE: Bead size exclusion
EpCAM: Epithelial cell adhesion molecule
FCM: Flow cytometry
FoC: Field of cancerization
FVS510: Fixable Viability Stain 510
HBSS: Hank’s Balanced Salt Solution
ME: Microenvironment
Pan-CK: Pan-cytokeratin
SECs: Squamous epithelial cells
TCPP: Tetra(4-carboxyphenyl)porphyrin
