# Supplementary material for: Detection of early-stage lung cancer in sputum using automated flow cytometry and machine learning
Source: Respir Res. 2023 Jan 21;24:23. doi: 10.1186/s12931-023-02327-3 (PMC9862555; doi:10.1186/s12931-023-02327-3)
Supplement: Supplementary file 1 — Additional file 1. Supplemental methods. Detailed description of automated flow cytometry data analysis and classifier development using generalized linear models. [file 12931_2023_2327_MOESM1_ESM.docx]

**SUPPLEMENTAL METHODS**

**1. Sample Processing Pipeline**

***Analysis Tubes*:**

- NIST beads
- Compensation tubes (one per fluorescence channel)
- Patient single-cell suspension (4 tubes):
  - Unstained as control for PE-anti-CD45, and TCPP
  - Isotype control stained with FVS510 (viability), PE-anti-CD45, PE-CF594 isotype, A488 isotype, FITC isotype
  - "Blood" stained with TCPP, FVS510, PE-anti-CD45, PE-CF594-anti-CD206, A488-anti-CD3, FITC-anti-CD66b, A488-anti-CD19 (note: A488 and FITC read out on the same channel resulting in a combined CD66b/CD19/CD3 signal)
  - "Epithelial" stained with TCPP, FVS510, PE-anti-CD45, PE-CF594-anti-EpCAM (epithelial cell adhesion molecule), A488-anti-PanCK (pan-cytokeratin)

***Analysis Steps for the LSRII Flow Cytometer [R Package(s)]*:**

**Note 1:** Steps for the Navios EX flow cytometer are essentially the same but adjusted for the different file format (i.e., LCM files for the Navios EX instead of FCS files for the LSRII) and detector sensitivity and dynamic range.

**Note 2:** Gate names are indicated in **BOLD UPPERCASE**.

**Note 3:** Temporary gate names are indicated in **bold lowercase**.

**Note 4:** Calculated threshold values are in ***bold italics***.

**Note 5**: Main package(s) used for current step are indicated by [square brackets].

**Note 6:** Some heuristic adjustments were needed to handle the wide range of sample composition and viability.

**2. Pseudocode:**

- For each tube, remove outlier events using time vs fluorescence channels. [flowCut]
- Use compensation tubes to automatically derive spillover matrix. [flowStats]
- Compensate fluorescence signals and transform to logicle scale. [flowWorkspace]
- Automatically find smallest NIST bead peak to set lower FSC-A threshold (***low***) to exclude bulk of debris. [openCyto]
- *Patient tubes*:
  - Keep events within the rectangular size gate, "**BSE**" (FSC-A: ***low***-2.5x10^5^, SSC-A: 0-2.5x10^5^). [flowWorkspace]
  - Apply a secondary "**NON-DEBRIS**" gate to **BSE** to exclude occasional unusual cell population high in FSC-H (>2x10^5^) and intermediate/high in SSC-H (>1x10^5^). [flowWorkspace]
  - Use a subset of cells most likely to contain the live singlets population of interest (dummy gating set) to set viability gate coordinates. This helps reduce artifacts and distortions from contaminating squamous epithelial cells (SECs) and other dead cells.
    - Set an automatic flowClust gate on **NON-DEBRIS** events using FSC-H vs SSC-H, "**hxh**", to exclude most of the contaminating SECs, restricting both channels to < 1x10^5^ and using a 0.99 quantile cutoff. [openCyto, flowClust]
      - If < 10% of events are retained in **hxh** (***low.viable = true***), relax channel restrictions to 1.5x10^5^ and quantile to 0.9.
    - Set an automatic singletGate "**singlets**" on **hxh** using FSC-A vs FSC-W channels and the "wider_gate=TRUE" setting. [openCyto, flowStats]
      - Force the upper right FSC-A coordinate to the same value as the lower right one to avoid cases were persisting SECs skew the gate downwards. [flowWorkspace]
    - Set an automatic tailGate "**VIABLE**" on **singlets** using the FVS510-A channel, restricting to min = 2, max = 3 (min and max on logicle scale), tolerance = 0.1. [openCyto]
      - If ***low.viable = true*** case, first restrict **singlets** events to SSC-A < 5x10^4^ and set smoothing parameter adjust = 1; [flowCore]
      - otherwise, first restrict **singlets** events to FSC-A < 5x10^4^ and set smoothing parameter adjust = 1.2. [flowCore]
  - Apply **VIABLE** gate determined from the dummy gating set to the full gating set **NON-DEBRIS** events. [flowWorkspace]
  - An automatic singletGate, "**SINGLETS**", set on **VIABLE** events gives good results in most cases but some samples still contain confounding events at this point.
    - SEC contamination can map close to larger viable cells which can result in a lower left coordinate < 0 or a lower right coordinate < the upper left one. In both cases, restrict **VIABLE** to SSC-A < 5x10^4^ before setting an automatic singletGate with "wider_gate = FALSE". [openCyto, flowStats]
    - In all cases, force the upper right FSC-A coordinate to the same value as the lower right one to avoid cases were persisting SECs skew the gate downwards. [flowWorkspace]
  - Apply **SINGLETS** gate to **VIABLE** events. [flowWorkspace]
  - When there are relatively few viable cells, the proportion of events retained in the **SINGLETS** gate in the gap between FVS510-A > 2.5 (logicle scale) and the viability cutoff can be substantial.
    - If "gap" population is > 10% of **SINGLETS**, force FVS501-A < 2.5 as viable cutoff.
      - Remove "**VIABLE**" from full gating set. [flowWorkspace]
      - Set "**VIABLE**" rectangleGate on **NON-DEBRIS** with FVS510-A < 2.5. [flowWorkspace]
      - Recalculate automatic singletGate "**SINGLETS**" as above. [openCyto, flowStats, flowWorkspace]
      - Apply **SINGLETS** gate to full gating set **VIABLE** events. [flowWorkspace]
- Write out full matrix of events retained by **SINGLETS** gate for each patient tube. These values along with patient metadata are the inputs to the Cancer/Non-Cancer classifier.

**3. Classifier Development Using Generalized Linear Models (GLMs) [R Base and Stats Packages]**

- Evaluate combinations of potential predictive factors:
  1. clinical parameters available for all samples
  2. quantized measurements using equally spaced or heuristically positioned breaks (3x3 and 4x4 grids per channel tested) from patient blood and epithelial samples (light and fluorescence), individually and in pairwise combinations
  3. quantized measurements as in #2 above minus background counts
  4. quantized fluorescence/log10(light scatter)
  5. frequency of specific subpopulations identified by manual analysis (Bederka et al., PLoS One. 2022;17:e0272069).
- Iterative step-forward and step-backward parameter inclusion in GLMs using distinct training and testing groups (roughly 2/3 training, 1/3 testing, randomly selected and non-overlapping). [stats::glm, stats::step]
- Evaluate GLMs based on the Akaike information criterion (AIC) which estimates prediction error and provides a comparator of model quality.
- Keep parameters retained repeatedly in different iterations of model testing with different combinations of potential predictive variables tested individually and in combinations to detect potential interactions.
- Control for over-fitting of the final model by repeated random sampling (n=10) of training/testing sets to verify predictive robustness independent of specific samples used to train the model.
- Validate processing pipeline and classifier predictions on samples not used in model building and testing.

**4. Supplementary References for Development Framework and Pipeline Library Dependencies**

**R v.3.6.2**

R Core Team (2019). R: A language and environment for statistical computing. R Foundation for Statistical Computing, Vienna, Austria. URL https://www.R-project.org/.

**Stats package step and glm functions**

Dobson A.J. (1990) An introduction to generalized linear models. London: Chapman and Hall.

Hastie T.J. and Pregibon D. (1992) Generalized linear models. Chapter 6 of Statistical Models in S eds J.M. Chambers and T.J. Hastie, Wadsworth & Brooks/Cole.

McCullagh P. and Nelder J.A. (1989) Generalized linear models. London: Chapman and Hall.

Venables W.N. and Ripley B.D. (2002) Modern applied statistics with S. New York: Springer.

**Bioconductor 3.9**

Orchestrating high-throughput genomic analysis with Bioconductor. Huber W., Carey V.J., Gentleman R., Anders S., Carlson M., Carvalho B.S., Bravo H.C., Davis S., Gatto L., Girke T., Gottardo R., Hahne F., Hansen K.D., Irizarry R.A., Lawrence M., Love M.I., MacDonald J., Obenchain V., Oleś A.K., Pagès H., Reyes A., Shannon P., Smyth G.K., Tenenbaum D., Waldron L., Morgan M. Nat Methods. 2015;12:115-121.

**library(CytoML)**

Jiang M., Spidlen J., Gopalakrishnan N., Hahne F., Ellis B., Gentleman R., Dalphin M., Le Meur N. and Purcell B. (2016) CytoML: A GatingML Interface for cross platform cytometry data sharing. R package version 1.11.11. URL https://github.com/RGLab/CytoML

**library(openCyto)**

Finak G., Frelinger J., Newell E.W., Ramey J., Davis M.M., Kalams S.A., De Rosa S.C., Gottardo R. OpenCyto: An open source infrastructure for scalable, robust, reproducible, and automated, end-to-end flow cytometry data analysis. PLoS Comput Biol. 2014;10:e1003806. version 1.23.5

**library(ggcyto)**

Van P., Jiang W., Gottardo R., Finak G. (2018) ggcyto: Next-generation open-source visualization software for cytometry. Bioinformatics URL https://doi.org/10.1093/bioinformatics/bty441. version 1.13.2

*Dependencies loaded:*

*ggplot2*

Wickham H. (2016) ggplot2: Elegant Graphics for Data Analysis. Springer-Verlag New York. version 3.2.1

*flowCore*

Ellis B., Haaland P., Hahne F., Le Meur N., Gopalakrishnan N., Spidlen J., Jiang M. and Finak G. (2019) flowCore: Basic structures for flow cytometry data. R package version 1.51.9.

*ncdfFlow*

Jiang M., Finak G., Gopalakrishnan N. (2019) ncdfFlow: A package that provides HDF5 based storage for flow cytometry data. R package version 2.31.6.

*RcppArmadillo*

Eddelbuettel D. and Sanderson C. (2014) RcppArmadillo: Accelerating R with high-performance C++ linear algebra. Computational Statistics and Data Analysis, Volume 71, March 2014, pages 1054-1063. URL http://dx.doi.org/10.1016/j.csda.2013.02.005. version 0.10.6

*BH*

Eddelbuettel D., Emerson J.W., and Kane M.J. (2021) BH: Boost C++ Header Files. R package version 1.75.0-0. URL https://CRAN.R-project.org/package=BH

*flowWorkspace*

Finak G. and Jiang M. (2011) flowWorkspace: Infrastructure for representing and interacting with gated and ungated cytometry data sets. R package version 3.33.10.

**Library(flowViz)**

Ellis B., Gentleman R., Hahne F., Le Meur N., Sarkar D., and Jiang M. (2019). flowViz: Visualization for flow cytometry. R package version 1.49.3.

*Dependency loaded:*

*lattice*

Deepayan S. (2008) Lattice: Multivariate Data Visualization with R. Springer, New York. ISBN 978-0-387-75968-5. version 0.20-38.

**Library(flowCut)**

Meskas J. and Wang S. (2019) flowCut: Precise and Accurate Automated Removal of Outlier Events and Flagging of Files Based on Time Versus Fluorescence Analysis. R package version 0.99.14.

**Library(gridExtra)**

Auguie B. (2017) gridExtra: Miscellaneous Functions for "Grid" Graphics. R package version 2.3. URL https://CRAN.R-project.org/package=gridExtra.

**Library(flowClust)**

Lo K., Hahne F., Brinkman R.R., and Gottardo R. FlowClust: a Bioconductor package for automated gating of flow cytometry data. BMC Bioinformatics. 2009;10:145.

Lo K., Brinkman R.R., and Gottardo R. Automated gating of flow cytometry data via robust model-based clustering, Cytometry A. 2008;73:321-332.

version 3.25.0

**Library(flowStats)**

Hahne F., Gopalakrishnan N., Khodabakhshi A.H., Wong C.-J. and Lee K. (2019) flowStats: Statistical methods for the analysis of flow cytometry data. R package version 3.43.9. URL http://www.github.com/RGLab/flowStats.
